# Facile and Green Fabrication of Porous Hydrogels Based on Gelatin Microsphere Porogens for 3D Immune Cell Culture

**DOI:** 10.3390/gels12060477

**Published:** 2026-05-29

**Authors:** Han Fu, Qiwen Yao, Shuai Tan, Yingming Wang, Aishun Jin

**Affiliations:** 1 Department of Immunology, School of Basic Medical Sciences, Chongqing Medical University, Chongqing 400010, China; f815819120@126.com (H.F.); 2023110070@stu.cqmu.edu.cn (Q.Y.); 2023110068@stu.cqmu.edu.cn (S.T.); wangyingming0908@cqmu.edu.cn (Y.W.); 2 Chongqing Key Laboratory of Tumor Immune Regulation and Immune Intervention, Chongqing 400010, China

**Keywords:** porous hydrogels, gelatin microspheres, viscoelasticity, interconnected pores, in situ encapsulation, cluster-forming growth, immune cells

## Abstract

Porous hydrogels are critical for tissue engineering and regenerative medicine, as they mimic the native extracellular matrix to support cell infiltration and mass transport. A common strategy for engineering pore structures involves the incorporation and subsequent removal of sacrificial porogen templates (e.g., crystals or microspheres). Although this approach offers excellent control over pore architecture, it often suffers from complex procedures and biosafety concerns arising from incomplete template removal. In this work, we present a simple, biocompatible, and versatile templating approach. By systematically investigating the coacervation parameters, we produced gelatin microspheres (GSs) with tunable diameters from 7 µm to 300 µm via a green, instrument-free, and scalable process. Using GSs of 20–160 µm as porogens, we obtained alginate hydrogels with adjustable viscoelasticity, stiffness, and pore sizes. We then validated two cell-loading strategies for bulk porous alginate hydrogels using immortalized human T (Jurkat) cells: (i) post-seeding into pre-formed pores supported high-density, long-term, and organized cell aggregates with >90% viability; (ii) in situ encapsulation (prior to pore formation) yielded >80% viability and preserved the cluster-forming growth characteristics of Jurkat cells. Moreover, composites of smaller GSs (7–20 µm) with alginate could be syringe-extruded into stable, sub-millimeter porous filaments, demonstrating the potential for 3D printing. Collectively, this work provides a promising platform for three-dimensional culture of immune cells.

## 1. Introduction

Hydrogels are extensively used as cell delivery vehicles in contexts such as tissue regeneration and adoptive cell therapy. An effective hydrogel must not only ensure sufficient nutrient and oxygen diffusion to maintain cell viability but also provide a structural microenvironment that supports specific cellular behaviors, such as spreading or aggregation. Nevertheless, concurrently optimizing fabrication methodology, mechanical properties, and biocompatibility remains a significant challenge [1,2,3].

Porous hydrogels are widely recognized as favorable platforms for supporting cell growth, and various strategies have been developed to introduce porosity. Among these approaches, the microsphere-based porogen templating method offers key advantages over phase separation, 3D printing, gas foaming, ice templating, and salt leaching. Unlike these methods, which often depend on organic solvents, costly equipment, or yield irregular pores, microsphere templating uses porogen microspheres that can be pre-fabricated in a clean manner, without specialized equipment, allowing precise control over pore size, shape, and distribution by simply adjusting microsphere morphology. Moreover, this method is compatible with a wide range of hydrogel materials and enables injectable in situ pore formation through microsphere degradation [4,5,6]. In addition to serving as pore-forming agents, microspheres can themselves provide multiple functionalities [7], such as drug delivery, vascular occlusion, and T cell activation, thereby offering dual benefits in the development of porous hydrogels constructed with microsphere-based porogens.

The choice and fabrication method of microspheres as porogen templates are crucial for the successful preparation of porous hydrogels, governing both process complexity and the biosafety of the final constructs. The microspheres must meet two critical criteria. First, the microspheres should exhibit broad material compatibility, along with a degradation profile that is controllable, operationally simple, and biocompatible. Second, the preparation process should be straightforward, environmentally benign, and amenable to scalable production. After a comprehensive evaluation of various gel microsphere systems [8,9], we focus our attention on gelatin-based microspheres [10]. The gelatin microspheres (GSs) degrade rapidly at 37 °C, without the need for any chemical reagents, and the gelatin molecules exhibit excellent biocompatibility for the majority of cells, including T cells that require stringent culture conditions. Microfluidic technology is currently the most extensively studied method for preparing GSs [11,12,13]. However, it faces limitations such as restricted material selection, low throughput, poor scalability, and high equipment costs. Although the oil-in-water emulsification method for preparing GSs can be carried out without specialized instrumentation [14,15,16,17], most protocols still involve the use of organic solvents to wash away the oil phase, which may introduce potential safety hazards and generate large amounts of organic waste. In 2019, 20 µm GSs prepared by a coacervation method were reported for the first time and used as a support material to facilitate 3D bioprinting [18,19]. Owing to the green and straightforward nature of the preparation process, these 20 µm GSs were subsequently employed to create porous structures within 3D-printed hydrogel filaments [20,21]. However, no report has yet described the preparation of GSs with a broader range of particle sizes using this method, and therefore their application scope in more diverse types of porous hydrogels remains limited.

Porous hydrogels are important for achieving three-dimensional (3D) cultures of immune cells, particularly T cells [6]. Upon antigen stimulation, T cells form activation clusters. The morphology of these clusters not only reflects the strength of the received stimulation but also involves intercellular ligand–receptor interactions (e.g., CTLA-4 and CD80) that govern T cell differentiation from a naïve to an effector state, or even to an exhausted state [22,23,24]. Therefore, fabricating porous hydrogels with optimized pore sizes can provide a suitable physiological microenvironment for T cell clustering, thereby supporting their proliferation and cytotoxic functions.

This study aims to comprehensively explore the preparation of GSs via a coacervation method and to establish a simple, green strategy for fabricating porous hydrogels using GSs as sacrificial porogen templates for future T cell immunomodulation. To this end, we first systematically investigated the influence of key parameters during the coacervation process on the morphology of GSs, successfully obtaining microspheres with controllable morphologies and tunable diameters. On this basis, we constructed a series of bulk porous hydrogels based on GSs and characterized their viscoelastic properties, confirming the presence of interconnected porous structures within the hydrogel networks. Furthermore, we explored two Jurkat cell loading strategies for the porous hydrogels, and the results demonstrated that the prepared porous hydrogels effectively supported cell growth in clusters and promoted the spatially organized distribution of cell aggregates. Finally, we validated the potential of this porous hydrogel fabrication method to be integrated with more advanced engineering approaches, such as bioprinting, in the future.

## 2. Results and Discussion

### 2.1. Fabrication of Morphologically Tunable Gelatin Microspheres

**Figure 1:**
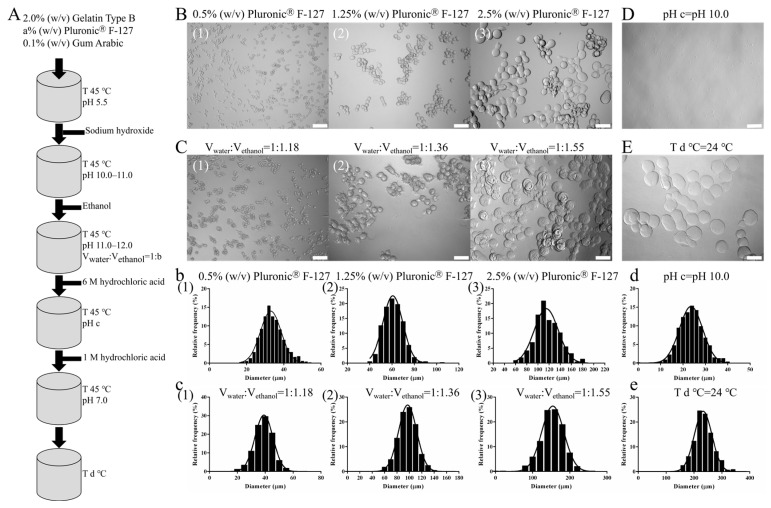
Preparation process and morphological variation patterns of GSs. (**A**) Schematic illustration of the preparation procedure and key adjustable parameters. (**B**) Morphological variation of GSs as a function of Pluronic^®^ F-127 concentration, and (**b**) the corresponding particle size distribution. Fixed parameters: V_water_:V_ethanol_ = 1:1.18, pH c = pH 9.4, T d °C = 19 °C. Pluronic^®^ F-127 concentrations: (1) 0.5% (*w*/*v*), (2) 1.25% (*w*/*v*), (3) 2.5% (*w*/*v*). Scale bar: 250 µm. (**C**) Morphological variation of GSs as a function of the V_water_:V_ethanol_ ratio and (**c**) the corresponding particle size distribution. Fixed parameters: 0.25% (*w*/*v*) Pluronic^®^ F-127, pH c = pH 9.4, T d °C = 19 °C. V_water_:V_ethanol_ ratios: (1) 1:1.18, (2) 1:1.36, and (3) 1:1.55. Scale bar: 250 µm. (**D**) Morphological image of GSs obtained by precisely controlling the V_water_:V_ethanol_ ratio and the pH of the mixture and (**d**) the corresponding particle size distribution. Fixed parameters: 0.25% (*w*/*v*) Pluronic^®^ F-127, V_water_:V_ethanol_ = 1:1.05, pH c = pH 10.0, T d °C = 19 °C. Scale bar: 250 µm. (**E**) Morphological image of GSs obtained by varying the cooling temperature during preparation and (**e**) the corresponding particle size distribution. Fixed parameters: 0.25% (*w*/*v*) Pluronic^®^ F-127, V_water_:V_ethanol_ = 1:1.36, pH c = pH 9.4, T d °C= 24 °C. Scale bar: 250 µm.

Gelatin (G) is a thermosensitive polymer that dissolves upon heating and forms a gel upon cooling, with gel stiffness increasing as the temperature decreases [25]. GSs were prepared by modulating the solubility of G through pH adjustments in a water/ethanol system, combined with controlled temperature variations. The final morphology of the GSs was governed by multiple processing parameters, including polymer concentration, ethanol content in the reaction mixture, the rate of pH change of the solution, and cooling temperature (Figure 1A). Among these, adjusting the initial pH to an alkaline range was the most critical step, as it ensured complete dissolution of the polymer and yielded a clear, transparent solution. This prevented premature precipitation of G upon ethanol addition, which would otherwise result in failed GS formation. Similar to the previously reported concentration-dependent increase in Pluronic^®^ F-127 micelle size [26], we observed that increasing Pluronic^®^ F-127 concentration from 0.5% to 2.5% (*w*/*v*) led to an approximately 2- to 3-fold increase in microsphere diameter (Figure 1B,b). Additionally, we observed that increasing the ethanol content accelerated polymer precipitation during pH downregulation, leading to a marked increase in both microsphere diameter and inter-microsphere adhesion (Figure 1C,c). At a V_water_:V_ethanol_ ratio of 1:1.05, the microspheres exhibited spherical to ellipsoidal morphologies, with an average diameter of 27 μm, and remained well separated (Figure 1D,d). As the ethanol proportion increased, microsphere diameters increased to approximately 160 μm, accompanied by increased particle stacking. This coalesced morphology of the GSs enabled the subsequent fabrication of porous hydrogels with interconnected pores. Notably, the concentration of hydrochloric acid (HCl) governed the pH reduction rate and consequently polymer precipitation. The pH was first adjusted to 9.0–10.0 using 6 M HCl, then fine-tuned to 7.0 with 1 M HCl. At a fixed V_water_:V_ethanol_ ratio of 1:1.05, the particle diameters decreased as pH c approached pH 10.0, without particle aggregation (Figure 1D,d). Moreover, raising the cooling temperature of the system produced larger microspheres up to 300 μm in diameter with irregular edges, likely due to looser chain entanglements at higher temperatures, resulting in more swollen structures (Figure 1E,e). Subsequent studies could further refine the morphology of GSs by exploring key parameters, including G concentration, molecular weight, and stirring rate.

The mean diameters and polydispersity indices of the GSs from three replicate batches prepared with each formulation are presented in Table S1. As shown, the diameter of the GSs ranged from approximately 7 µm to 300 µm, and the changes in diameter in response to variations in Pluronic^®^ F-127 concentration, ethanol volume, pH, and cooling temperature were consistent. The numerical fluctuations were mainly attributable to manual pH adjustment, which may affect the rate of pH decrease, and more importantly, to the fact that the cooling of the GSs was performed at room temperature without precise control of the cooling rate. To achieve better control over microsphere morphology and reproducibility, a more sophisticated setup for precisely controlling the preparation process could be employed.

A major drawback of current solvent-based methods for GSs preparation is the inconsistency of solvents used across studies and the lack of a standardized approach to produce microspheres of different diameters [14,15,16,17]. We tested two solvent-based methods: olive oil produced aggregated microspheres, while paraffin oil produced only irregular gelatin sheets (Figure S1). A comparison with our established method revealed two key differences. First, reagent waste: our method used only water and ethanol (universally available, consistent properties), whereas solvent-based methods required oil-phase solvents with varying physicochemical properties, which must be empirically selected and can lead to waste. Second, process safety: our 100 mL system used three PBS washes (about 200 mL total) and yielded non-cytotoxic products. The solvent-based 10 mL system required four isopropanol washes (40 mL total), followed by three PBS washes (30 mL total), increasing the washing steps and the risk of organic solvent contamination.

### 2.2. Characterization of the Mechanical Properties and Internal Structure of Gelatin Microspheres-Based Bulk Hydrogels

We further investigated the rheological properties of hydrogels constructed from GSs. Under amplitude sweeps performed at 1 Hz with strains below 10%, the storage modulus (*G′*) of the GSs exceeded the loss modulus (*G″*), indicating that the GSs behaved as a colloid with moderate mechanical strength. Upon increasing the strain, the GSs underwent a transition to a fluid-like state. Accordingly, both the frequency sweep and shear stress-shear rate curves demonstrated that the GSs exhibited yield-stress fluid behavior (Figure S2). Based on this rheological property, GSs have been employed as a support bath to facilitate the fixation of soft material-based gel filaments during extrusion-based bioprinting [18].

**Figure 2:**
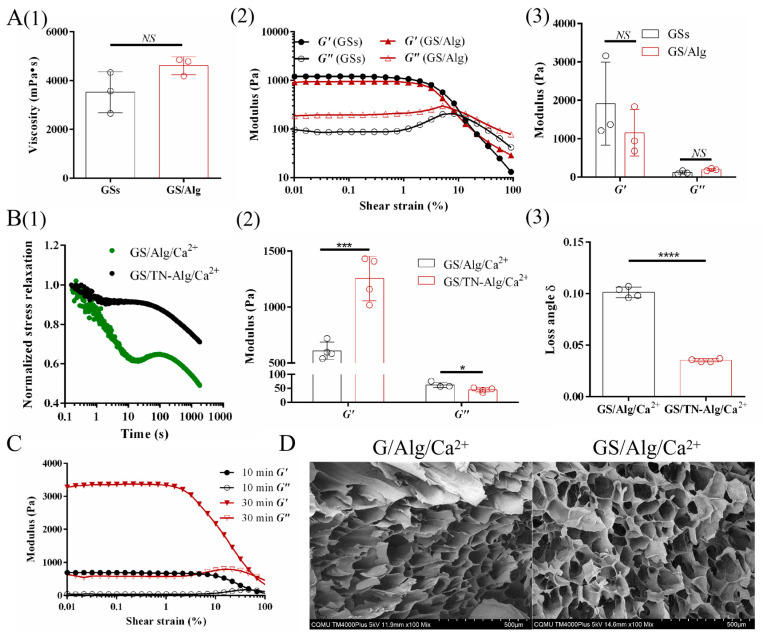
Rheological properties and internal morphology of GSs and GS-based porous hydrogels. (**A**) (1) Comparison of viscosity between GSs and GS/Alg composites; (2) shear strain sweep curves of GSs and GS/Alg composites; and (3) the corresponding comparison of storage modulus (*G′*) and loss modulus (*G″*). (**B**) (1) Stress relaxation curves of viscous (ionic crosslinking, GS/Alg/Ca^2+^) and elastic hydrogels (ionic and covalent Nb-Tz crosslinking, GS/TN-Alg/Ca^2+^); (2) and (3) comparison of *G′*, *G″*, and loss factor (tan δ) for GS/Alg/Ca^2+^ and GS/TN-Alg/Ca^2+^ hydrogels, respectively. (**C**) Shear strain sweep curves of GS/Alg/Ca^2+^ hydrogels under Ca^2+^ crosslinking conditions for 10 min and 30 min. (**D**) Scanning electron microscopy (SEM) images of G/Alg/Ca^2+^ and GS/Alg/Ca^2+^ hydrogels. Scale bar: 500 µm. Statistical analysis was performed using an unpaired *t*-test. * *p* < 0.05, *** *p* < 0.001, **** *p* < 0.0001. *NS:* no significant difference (*p* > 0.05).

Calcium-crosslinked alginate (Alg) hydrogels are currently the most common ionically crosslinked gels in 3D cell culture. The instability of the ionic crosslinking network leads to rapid gel degradation [27], an advantageous feature for the in vivo delivery of cells requiring fast effector functions, such as CAR-T cells [28,29]. Moreover, Alg molecules can be chemically modified via their carboxyl groups to form covalently crosslinked, stable hydrogels. Ionically and covalently crosslinked Alg hydrogels are often compared to evaluate how the mechanical environment influences the maintenance of immune cell biological activity [30,31]. Therefore, we next constructed several GS-based alginate hydrogels to assess the impact of GSs on the mechanical properties and internal morphology of Alg hydrogels. The composite formed by integrating GSs with Alg, a viscous polymer, retained moderate mechanical strength; however, the inclusion of Alg led to a reduced *G′*, elevated *G″*, and increased viscosity relative to the GS-only group (Figure 2A). Previous reports demonstrated that calcium (Ca^2+^)-crosslinked Alg hydrogels behaved predominantly as viscous materials, whereas the incorporation of covalent “click” crosslinking via tetrazine (Tz)-norbornene (Nb) chemistry introduced elastic characteristics [30]. We further explored whether viscous and elastic hydrogels constructed from GSs retained these distinct rheological features. The successful synthesis of Tz-Alg and Nb-Alg was verified by ^1^H NMR spectra (Figure S3). Both the TN-Alg composite (comprising Tz-Alg and Nb-Alg) and unmodified Alg were separately blended with GSs, crosslinked with Ca^2+^, and then compared in terms of their rheological properties. Figure 2B demonstrated that elastic GS/TN-Alg/Ca^2+^ hydrogels displayed slower stress relaxation, along with lower *G″* and loss factor (tan δ). Moreover, the secondary crosslinking significantly enhanced the *G′* of GS/TN-Alg/Ca^2+^ hydrogels, thereby strengthening their mechanical integrity. These findings confirmed that GS-based hydrogels retained both the well-established viscous and elastic characteristics, depending on their crosslinking design [32,33,34]. Consistent with prior reports on Alg hydrogels [35], the mechanical properties of GS/Alg/Ca^2+^ porous constructs were Ca^2+^-dependent, showing a 6-fold enhancement after 30 min of crosslinking relative to 10 min (Figure 2C).

Then, we prepared Ca^2+^-crosslinked gelatin/alginate hydrogels (G/Alg/Ca^2+^) and compared their internal structure with that of GS/Alg/Ca^2+^ hydrogels. As shown in Figure 2D, G/Alg/Ca^2+^ group exhibited stacked, hollow channel-like structures with limited pore interconnectivity. In contrast, GS/Alg/Ca^2+^ group exhibited densely distributed and interconnected pores-an architectural feature also observed in GS/TN-Alg/Ca^2+^ constructs (Figure S4A). Notably, GS/Alg/Ca^2+^ hydrogels fabricated with larger-diameter GSs (140–160 μm) exhibited markedly increased pore sizes compared to those prepared with 20–40 μm microspheres, demonstrating that GSs can be utilized to engineer porous hydrogels with readily tunable pore sizes (Figure S4C). Additionally, GS/methacrylated Alg (MA-Alg) composites were employed to fabricate blue light (BL)-crosslinked hydrogels (GS/MA-Alg/BL). Interestingly, although a certain cross-section of the GS/MA-Alg/BL sample displayed a laminar structure similar to that of G/Alg/Ca^2+^ hydrogels, interconnected pores were observed between adjacent layers (Figure S4B). Freeze-drying methods influence the pore structure of hydrogels. We compared the morphology of GS/Alg/Ca^2+^ hydrogels after slow freezing at −80 °C for 4 h versus flash freezing in liquid nitrogen for 8 min, followed by lyophilization. Macroscopically, slow freezing at −80 °C better preserved the cylindrical shape of the hydrogels, whereas liquid nitrogen treatment caused visible collapse. Internally, the slow-frozen samples had a disordered pore arrangement, with round-shaped pores, while the liquid nitrogen-frozen samples exhibited a more organized, compact pore architecture with rectangular-shaped pores (Figure S5) [36]. Contrary to the reported trend that pore size decreases at lower freezing temperatures [37], we found no significant difference in pore diameter between slow freezing (−80 °C) and flash freezing (liquid nitrogen): 92.95 ± 34.40 µm vs. 98.70 ± 36.47 µm. This discrepancy may be attributed to the presence of pre-existing large pores within our hydrogels, rather than only ice crystal-induced pores. Green fluorescence-labeled Alg (GF-Alg) was then used to prepare GS/GF-Alg/Ca^2+^ hydrogels, and a 3D fluorescence reconstruction image revealed a layered porous architecture (Figure S6(1)). Quantitative image analysis yielded a porosity of 52%. After incubation in culture medium containing Cy3-labeled gelatin for 12 h, approximately 58% of the pores exhibited Cy3-gelatin penetration (Figure S6(2)). The successful preparation of porous hydrogels with interconnected pores in this work relies on retaining only the microsphere pellet after centrifugation of the GS/Alg mixture, thereby achieving extremely high microsphere loading within the gel matrix. This strategy is feasible for two reasons: (1) GSs are highly compatible with Alg and do not interfere with Alg gelation; and (2) GSs possess excellent biosafety, eliminating the need for any exogenous chemicals to facilitate their degradation.

To evaluate time-dependent pore formation, calcium-crosslinked hydrogels composed of Cy3-labeled GSs (Cy3-GS) and Alg were prepared at 4 °C and incubated at 37 °C. The release kinetics of Cy3 into the culture medium were monitored over time. As shown in Figure S7A, the Cy3 signal increased rapidly during the first 4 h, after which the increase gradually slowed. The slight increase observed between 12 h and 24 h was largely attributable to medium evaporation, which concentrated the Cy3 signal. These results indicated that the GSs degraded rapidly within the first 4 h. Comparison of the internal morphology of Cy3-GS/Alg/Ca^2+^ constructs at 0 and 4 h revealed that at 0 h, the Cy3 signal was confined within the GSs, whereas after 4 h, the original positions of the GSs became translucent, and the Cy3 signal was released into the Alg matrix (Figure S7B). Moreover, the distribution of the Cy3 signal suggested that while some gelatin was released into the external medium, a portion remained within the hydrogel, which may influence cellular biological functions.

Although we have obtained rheological data for the GS-based Alg hydrogel, we still lack evaluation of other key mechanical parameters, such as compressive modulus, elastic modulus, and Young’s modulus. Therefore, it remains challenging to comprehensively assess how the addition and subsequent degradation of GS porogens affect the mechanical properties of the hydrogel, as well as to fully evaluate the potential changes in cellular biological behavior in response to the alterations in the hydrogel’s mechanical properties. Future work could test the GS-based method with hydrogels that have better mechanical properties, more complex structures, or advanced processing techniques [38,39].

### 2.3. Assessing the Long-Term Support of High-Density Cell Viability in Gelatin Microspheres-Based Hydrogels

We evaluated the mechanical properties and internal morphology of ionically crosslinked and covalently crosslinked GS-based porous Alg hydrogels in Section 2.2. Next, we investigated their characteristics in 3D cell culture.

**Figure 3:**
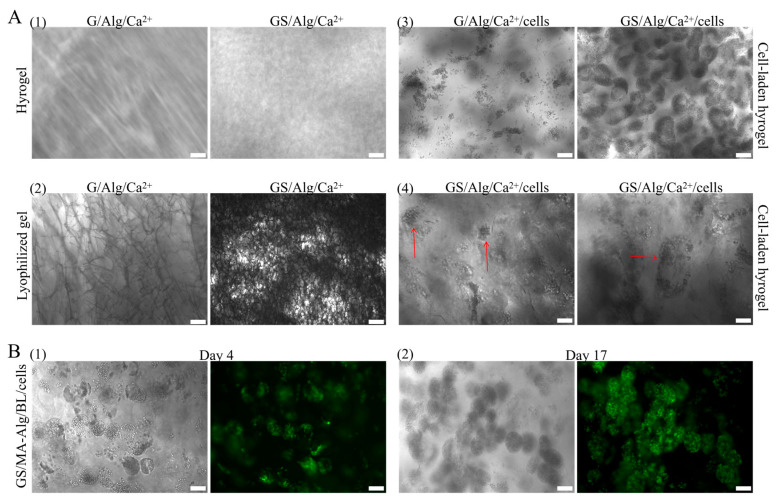
Evaluation of the capability of GS/(modified) Alg composite porous hydrogels for cell loading and support of cluster-forming cell growth. (**A**) (1) Optical microscopy images of the G/Alg/Ca^2+^ and GS/Alg/Ca^2+^ hydrogels. Scale bar: 100 µm. (2) Optical microscopy images of the G/Alg/Ca^2+^ and GS/Alg/Ca^2+^ constructs after lyophilization. Scale bar: 250 µm. (3) Jurkat cells loaded into lyophilized G/Alg/Ca^2+^ and GS/Alg/Ca^2+^ constructs, with cell distribution observed by optical microscopy. Scale bar: 100 µm. (4) High-magnification optical microscopy image showing the distribution of Jurkat cells within the GS/Alg/Ca^2+^ hydrogels. Red arrows indicate cell clusters encapsulated within the pores (left panel) and those distributed along the pore edges (right panel). Scale bar: 50 µm. (**B**) Optical microscopy images of Jurkat cell distribution within GS/MA-Alg/Ca^2+^ hydrogels. Scale bar: 100 µm.

We investigated the feasibility of using freeze-dried GS-based Alg hydrogels as scaffolds to load Jurkat cells expressing enhanced green fluorescent protein (EGFP), and examined cell distribution and viability. Optical microscopy revealed that the surface of G/Alg/Ca^2+^ hydrogels exhibited parallel wrinkled striations, while the lyophilized samples displayed irregularly sized pores covered by a veil-like layer. In contrast, the GS/Alg/Ca^2+^ hydrogels displayed large, overlapping circular pores on the surface, and upon freeze-drying, exhibited densely packed pores with relatively uniform dimensions (Figure 3A(1,2)). Jurkat cells were seeded onto the surface of the freeze-dried hydrogels, and after several minutes of absorption, the constructs were immersed in culture medium to allow rehydration and swelling. At 3 days post-seeding, optical microscopy showed sparse, randomly distributed cell aggregates on the G/Alg/Ca^2+^ hydrogels. In contrast, the GS/Alg/Ca^2+^ hydrogels supported the formation of numerous, well-ordered cell clusters, which exhibited either solid or hollow morphologies (Figure 3A(3)). Higher magnification imaging revealed cell aggregates both within the pores and along their perimeters (Figure 3A(4)). Parallel experiments were conducted using lyophilized photo-crosslinked GS/MA-Alg/BL hydrogels, which possessed a porous structure similar to that of GS/Alg/Ca^2+^ hydrogels (Figure S4B). Following identical cell loading procedures, the Jurkat cells within these gel pores proliferated over time, progressing from sparse, thin clusters to dense, robust aggregates (Figure 3B).

**Figure 4:**
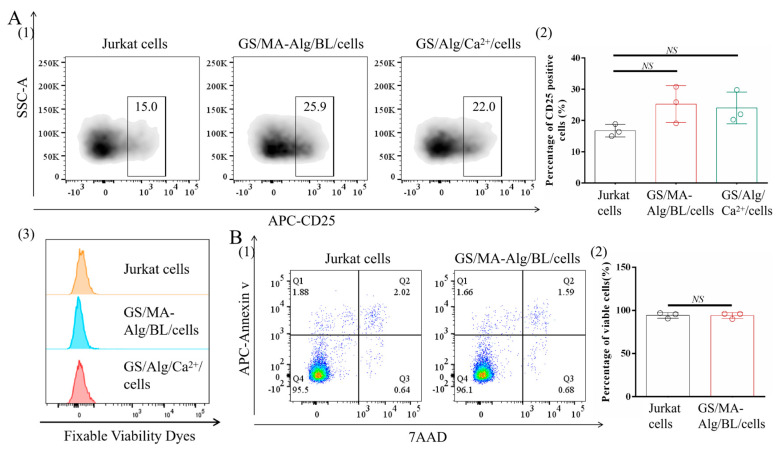
Evaluation of the viability of Jurkat cells cultured within GS/Alg/Ca^2+^/cells hydrogels. (**A**) Jurkat cells cultured for 3 days in GS/Alg/Ca^2+^ hydrogels or for 6 days in GS/MA-Alg/Ca^2+^ hydrogels were compared with normally cultured Jurkat cells following 24 h stimulation with CD3/CD28 magnetic beads. (1) Flow cytometry analysis of CD25 expression levels; (2) statistical plots of CD25 expression levels; (3) flow cytometry analysis of cell viability. (**B**) Flow cytometry analysis (1) and statistical plots (2) of apoptosis and necrosis levels in Jurkat cells harvested after 17 days of culture in the GS/MA-Alg/Ca^2+^ hydrogels, compared with normally cultured Jurkat cells. APC-Annexin V: early apoptosis; 7-AAD: late apoptosis/necrosis. Quadrant interpretation: Q1, early apoptotic cells; Q2, late apoptotic/necrotic cells; Q3, necrotic cells; Q4, viable cells. Statistical analysis was performed using an unpaired *t*-test. *NS:* no significant difference (*p* > 0.05).

After designated culture periods (3 days for GS/Alg/Ca^2+^/cells, 6 days for GS/MA-Alg/BL/cells), the Jurkat cells were harvested from the hydrogels and stimulated for 24 h with CD3/CD28 activation beads. The selection of different detection time points for the two groups of hydrogels was primarily based on the stability of ionically crosslinked versus covalently crosslinked hydrogels during culture. A flow cytometry analysis of Jurkat cells stained with Fixable Viability Dyes demonstrated that cell viability in the GS/Alg/Ca^2+^/cells and GS/MA-Alg/BL/cells groups was comparable to that of normally cultured Jurkat cells, accompanied by minimal dead cell counts (Figure 4A(3)). In addition, approximately 20% of the Jurkat cells expressed CD25, a T cell activation marker, representing a slight elevation compared to conventionally cultured Jurkat cells (Figure 4A(1,2)). These results confirm that the Jurkat cells cultured in hydrogels remained functionally intact. Notably, the Jurkat cells cultured within GS/MA-Alg/BL/cells hydrogels for up to 17 days maintained a viability above 90%, underscoring the long-term biocompatibility of the GS-based scaffolds (Figure 4B). It is generally accepted that hydrogels with greater mechanical strength tend to possess smaller pore sizes [40,41], which in turn hinders the absorption of cells by freeze-dried samples in the manner discussed above. However, GS/(modified) Alg-based hydrogels have the potential to reconcile the need for robust mechanical properties with the capacity to absorb large numbers of cells through their porous architecture.

### 2.4. Assessment of in Situ Cell Encapsulation in Ca^2+^-Crosslinked Gelatin Microspheres/Alginate Hydrogels

**Figure 5:**
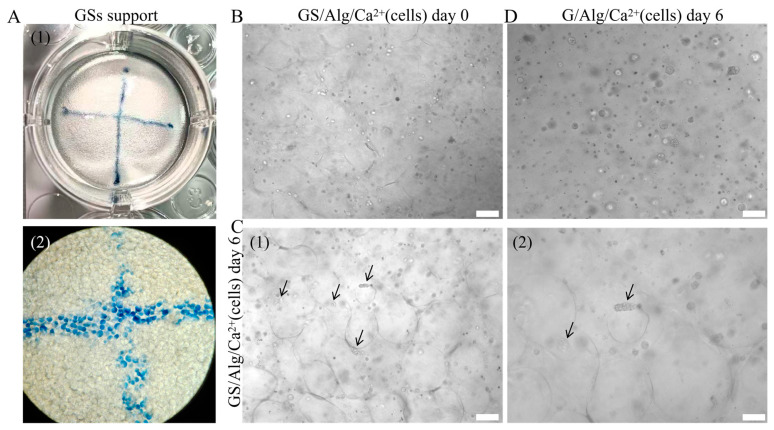
Evaluation of the capability of GS/Alg composite-based porous hydrogels for in situ cell encapsulation and the support of cluster-forming cell growth. (**A**) Visual representation of GSs serving as supports for the cross-shaped extrusion molding of the trypan blue-stained Alg solution. (1) Macroscopic image; (2) Optical microscopy image. (**B**) Distribution of the Jurkat cells encapsulated within the porous GS/Alg/Ca^2+^ (cells) hydrogels at day 0. Scale bar: 100 µm. (**C**) Distribution of Jurkat cells encapsulated within the GS/Alg/Ca^2+^ (cells) hydrogels at day 6. Scale bars: (1) 100 µm and (2) 50 µm. (**D**) Distribution of the Jurkat cells encapsulated within the G/Alg/Ca^2+^ (cells) hydrogels at day 6. Scale bar: 100 µm. Black arrows indicate Jurkat cells growing in clusters.

Figure 5A illustrates that GSs could serve as a support matrix to facilitate the extrusion of trypan blue-stained viscous Alg solution into defined geometries, such as a cross pattern. This capability inspired us to hypothesize that different cell types could be selectively encapsulated within distinct regions: Alg gel domains (e.g., the cross region) and GS-based gel domains (e.g., the quadrant areas), thereby enabling precise spatial arrangement of cells in 3D constructs. To explore this possibility, we selected the immortalized human T cell line (Jurkat cells), which exhibits cluster-forming growth characteristics, as a model to evaluate whether GS/Alg/Ca^2+^ hydrogels could encapsulate Jurkat cells in situ and support their viability. As shown in Figure 5B, the Jurkat cells were uniformly distributed within the porous gel network immediately after encapsulation. After 6 days in culture, two distinct distribution patterns emerged within the GS/Alg/Ca^2+^ (cells) group: loose cell clusters localized within the gel pores and dense cell aggregates forming between the pores (Figure 5C). In contrast, the Jurkat cells cultured in the conventional G/Alg/Ca^2+^ (cells) group predominantly formed compact spheroids (Figure 5D). To further verify the internal structure of the GS/Alg/Ca^2+^ (cells) hydrogel, Cy3-GS/GF-Alg/Ca^2+^ hydrogels loaded with DAPI-labeled Jurkat cells were prepared and stored at 4 °C (Figure S6(3)). Observations revealed that some cells were dispersed within the GF-Alg matrix, while others were localized at the interface between Cy3-GS and GF-Alg. After incubation at 37 °C for 48 h, the Cy3-GS signal decreased, leaving some residual signal at pore margins, and a portion of DAPI-labeled cells remained within the pores (Figure S6(4)). These observations suggest that the GS-based composite gel more effectively preserves the natural cluster-forming growth characteristics of Jurkat cells.

Although we observed Jurkat cell aggregates in the GS/Alg/Ca^2+^ (cells) hydrogel, the proportion of aggregates was relatively low. The main reasons were as follows: First, unlike the preparation method for GS/Alg/Ca^2+^/cells (where excess Alg was discarded to retain a high proportion of GSs), the GS/Alg/Ca^2+^ (cells) preparation involved mixing the GS–Jurkat cell mixture with an equal volume of Alg without discarding any Alg to avoid cell loss. This resulted in a reduced proportion of GSs within the hydrogel, causing cells to be predominantly distributed in the Alg regions, which made it difficult for them to form aggregates within the voids created by the degradation of GSs. Second, compared with GS/Alg/Ca^2+^/cells, cell viability in GS/Alg/Ca^2+^ (cells) was slightly lower, leading to a reduced number of cells available for aggregate formation. Third, during imaging, only a single focal plane of the hydrogel could be captured; thus, the distribution of Jurkat cell aggregates across multiple planes was not observed.

**Figure 6:**
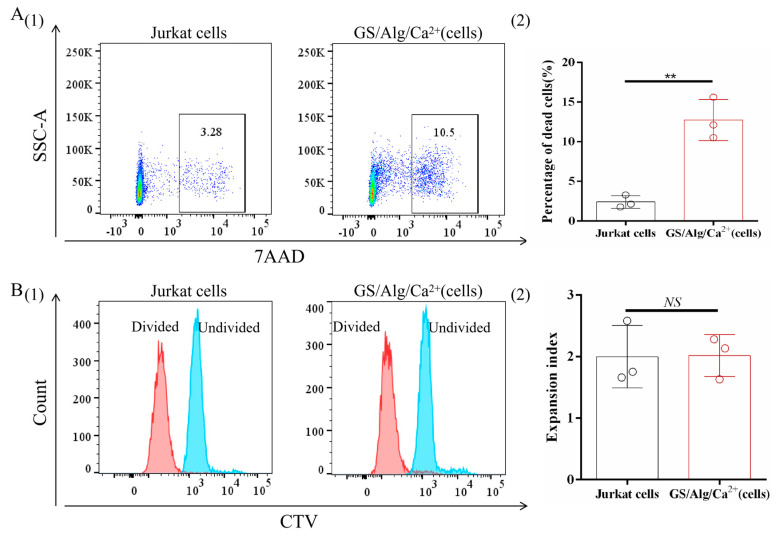
Evaluation of the viability of Jurkat cells encapsulated in situ within GS/Alg/Ca^2+^ (cells) hydrogels. (**A**) Flow cytometry analysis (1) and statistical plots (2) of the proportion of necrotic Jurkat cells after 3 days of culture in the GS/Alg/Ca^2+^ (cells) hydrogels, compared with normally cultured Jurkat cells. (**B**) Flow cytometry analysis (1) and statistical plots (2) of the cell proliferation index of Jurkat cells after 3 days of culture in the GS/Alg/Ca^2+^ (cells) hydrogels, compared with normally cultured Jurkat cells. 7-AAD: late apoptosis/necrosis. CTV: cell proliferation indicator dye. Statistical analysis was performed using an unpaired *t*-test. ** *p* < 0.01. *NS:* no significant difference (*p* > 0.05).

Following 3 days of in-gel culture, the harvested Jurkat cells were stained with 7-Aminoactinomycin D (7-AAD), revealing a viability exceeding 80% (Figure 6A). Additionally, cells labeled with the CellTrace^TM^ Violet (CTV) displayed a comparable decay pattern of fluorescence intensity to that of conventionally cultured Jurkat cells, indicating that their proliferative capacity remained unaffected (Figure 6B). Collectively, these findings demonstrate that the GS/Alg/Ca^2+^ (cells) hydrogels not only support Jurkat cell viability but also maintain their proliferative potential, highlighting their promise as bioinks for spatially organized 3D cell culture and bioprinting applications.

### 2.5. Morphological Characterization of Extruded GS-Based Hydrogel Fibers at the Micron Scale

**Figure 7:**
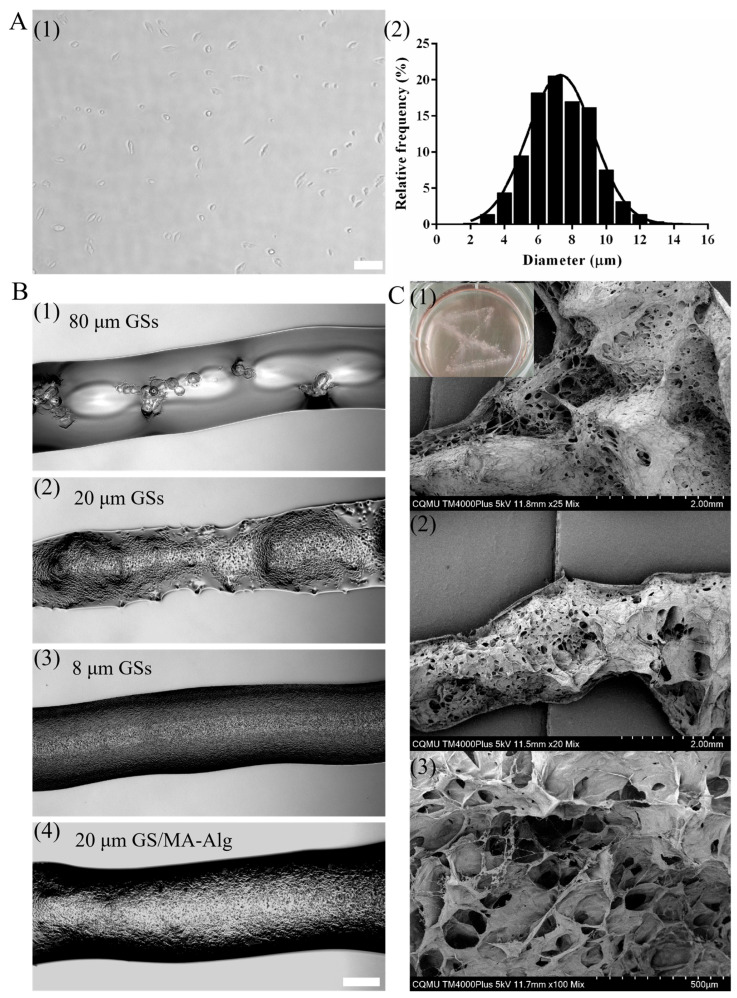
Fabrication and structural characterization of Alg gel filaments based on small-sized GSs. (**A**) (1) Morphology of ultra-small GSs under optical microscopy and (2) the corresponding diameter distribution histogram. Scale bar: 50 µm. (**B**) Optical microscopy images of filaments extruded from GSs of different sizes and from the composite of 20 µm GSs and MA-Alg. Scale bar: 250 µm. (**C**) Internal structure of blue light-cured extruded hydrogel filaments based on GS/MA-Alg: (1) structure at a turning point; (2) and (3) show structures along straight segments at different magnifications. Scale bars: (1) 2.00 mm, (2) 2.00 mm, (3) 500 µm.

In the previous section, we first demonstrated that GSs can serve as a support material to facilitate the extrusion of Alg hydrogel filaments, thereby potentially enabling the ordered arrangement of different cells, both within the GS-based bulk hydrogel and within the Alg hydrogel filaments. On this basis, we further hypothesized that porous fiber-grade hydrogels based on GSs could be fabricated via extrusion to increase the complexity of multi-cell patterning in future work. Next, we focused on the preparation of porous Alg hydrogel microfibers incorporating GSs. First, we prepared ultra-small GSs with an average diameter of approximately 8 µm (Table S1) using a water-to-ethanol volume ratio of 1:1 and a pH c of 10.0 (Figure 1), which exhibited spherical, ellipsoidal, and fusiform morphologies (Figure 7A). GSs of varying diameters were extruded into filaments through a 27G syringe, and their distribution within the filaments was examined (Figure 7B). As shown in Figure 2A, the GSs displayed Bingham plastic rheological behavior during extrusion: under high shear stress (e.g., when passing through the needle), they flowed as a viscous fluid; upon exiting the needle under low shear stress, they behaved as a solid, yielding gel filaments that did not collapse or leak [18,42]. Only the filaments extruded from 8 µm GSs exhibited homogeneous microsphere distribution and smooth filament morphology. With increasing microsphere diameter, the distribution became heterogeneous, and filament smoothness was compromised. Extrusion of a 1:1 (*v*/*v*) mixture of 20 µm GSs and 6% (*w*/*v*) MA-Alg resulted in a relatively even microsphere distribution, albeit with an increase in filament width. This mixture was further extruded using a 24G syringe into a structure consisting of two triangles placed tip-to-tip, followed by photo-crosslinking, removal of GSs at 37 °C, and freeze-drying to observe the internal architecture (Figure 7C). The results revealed porous structures at both the turning points and the straight segments of the filaments. Using an extrusion method, we prepared EGFP-Jurkat cell-loaded GS/MA-Alg/BL hydrogel microfibers (Figure S8). After 21 h of culture, both cell aggregates and EGFP fluorescence were observed within the fibers, demonstrating favorable cell viability. Although 3D printing using 20 µm GSs has already been reported [20,21], our ultra-small microspheres may offer further improvements in printing resolution.

## 3. Conclusions

In this work, we developed and systematically evaluated a novel strategy for constructing porous hydrogels using GSs as sacrificial porogen templates. The porogen templating approach offers notable advantages, including high controllability over pore shape and size (dependent on the design of porogens such as crystals or microspheres) and broad applicability across diverse material systems. However, its limitations include: (1) the need for pre-fabrication of templates, which increases process complexity; (2) poor pore interconnectivity, typically resulting in isolated pores; and (3) incomplete porogen removal, which may require toxic solvents. Our proposed method overcomes or alleviates these drawbacks. First, we established a green and straightforward protocol for the preparation and washing of GSs, enabling scalable production and reducing the template fabrication time. Second, owing to the high compatibility of GSs with various materials and the fact that gelatin degradation does not rely on any exogenous chemicals, the loading capacity of microspheres within the gel system can be increased, thereby ensuring enhanced pore connectivity. Third, the excellent biocompatibility of gelatin allows its degradation products to be utilized by cells, offering dual benefits. Leveraging these advantages, we achieved two modes of cell encapsulation, and the resulting porous hydrogels ranged from bulk constructs to gel filaments of several hundred micrometers.

In this work, Alg hydrogels were selected as the model system. For cells requiring long-term culture, the unstable mechanical properties of Alg hydrogels could lead to the loss of porous structure and reduced support for cells. However, the porous Alg hydrogel system may be well suited for T cells that favor short-term culture. The typical in vitro proliferation cycle of T cells is 14 days, and we have experimentally demonstrated that the pores in Alg hydrogels remain intact after 17 days of in vitro culture. Moreover, upon in vivo delivery, the degradation of Alg hydrogels can fulfill the biological requirement for rapid T cell release to exert their cytotoxic functions. Future efforts could focus on constructing porous hydrogels from a broader range of biomaterials or engineered variants with improved mechanical properties, thereby expanding the application scope of the method established in this work. This work will open new avenues for the development of porous hydrogels that combine robust mechanical performance with biomimetic functionality.

## 4. Materials and Methods

### 4.1. Materials

Gelatin Type B, gum Arabic, and alginate (Alg) were obtained from Sigma-Aldrich (St. Louis, MO, USA). Methacrylated alginate (MA-Alg) and photoinitiator LAP (lithium phenyl-2,4,6-trimethylbenzoylphosphinate) were obtained from Stemeasy Biotechnology, Inc. (Jiangyin, China). Pluronic^®^ F-127, Cyanine3 NHS ester (BF4), liquid paraffin, green fluorescence-labeled sodium alginate (GF-Alg), and (4-(1,2,4,5-tetrazin-3-yl)phenyl)methanamine hydrochloride (Tz) were obtained from Aladdin Biochemical Technology Co., Ltd. (Shanghai, China). 5-(aminomethyl)bicyclo[2.2.1]hept-2-ene (Nb) was obtained from Tokyo Chemical Industry (Tokyo, Japan). *N*-hydroxysuccinimide (NHS), *N*-(3-Dimethylaminopropyl)-*N′*-ethylcarbodiimide hydrochloride (EDC), and 2-morpholinoethanesulfonic acid (MES) were purchased from Macklin Biochemical Co., Ltd. (Shanghai, China). APC-Annexin V, 7-Aminoactinomycin D (7-AAD), APC-anti-human CD25 antibody, and the Zombie NIR^TM^ Fixable Viability Kit were obtained from BioLegend, Inc. (San Diego, CA, USA). CellTrace^TM^ Violet stain was obtained from Invitrogen (Carlsbad, CA, USA). CD3/CD28 magnetic beads were purchased from Miltenyi Biotec (Bergisch Gladbach, Germany). Olive oil was purchased from Aceites Borges Pont company (Reus, Spain). 

### 4.2. Cell Lines

Jurkat (Clone E6-1, ATCC, TIB-152) was cultured in RPMI media supplemented with 10% (*v*/*v*) fetal bovine serum (Excell Bio, Shanghai, China) and 1% (*v*/*v*) penicillin/streptomycin (Gibco, Waltham, MA, USA). A Jurkat cell line stably expressing enhanced green fluorescent protein (EGFP) was established via lentiviral transduction using an EGFP-encoding plasmid purchased from Miaoling Biotechnology Co., Ltd. (Wuhan, China).

### 4.3. Material Synthesis

Alg was functionalized via covalent conjugation of either Tz (Tz-Alg) or Nb (Nb-Alg), following previously established protocols [43]. Briefly, Alg was dissolved at 1% (*w*/*v*) in MES buffer (0.1 M MES, 0.3 M sodium chloride (NaCl), pH 6.5). Subsequently, NHS and EDC were added at a 5-fold molar excess relative to the carboxylic acid groups of the Alg. Either Nb or Tz was added at a concentration of 1 mmol per gram of Alg. The resulting reaction mixture was stirred at room temperature for 16 h. Following the reaction, the products were purified by dialysis (molecular weight cutoff (MWCO) 3500 Da) and lyophilized for further use.

### 4.4. Hydrogel Preparation

Figure S9 depicts the main workflow for sample preparation.

**Gelatin microspheres (GSs):** GSs were fabricated via a complex coacervation process. Briefly, gelatin Type B (G), Pluronic^®^ F-127, and gum Arabic were dissolved in a water/ethanol mixture at 45 °C. The pH of the solution was subsequently adjusted to 7.0 using HCl. The mixture was then allowed to cool gradually to room temperature with continuous stirring overnight. The resulting slurry was washed three times using phosphate-buffered saline (PBS, pH 7.4) by centrifugation at 1000× *g* for 10 min each cycle. The diameters of GSs from three replicate batches prepared with each formulation were statistically analyzed and expressed as mean diameter ± standard deviation. The polydispersity index (PDI) was calculated as the square of the standard deviation divided by the square of the mean diameter.

**Cy3-GS:** Cy3-NHS ester was co-incubated with 80 µm GSs overnight, then washed four times with 0.9% (*w*/*v*) NaCl solution and stored at 4 °C.

**Cy3-G:** Cy3-GSs were dialyzed overnight, dissolved at 45 °C, and lyophilized.

**GS/Alg composite:** A 2% (*w*/*v*) Alg solution was prepared and subsequently mixed thoroughly with GSs at a volume ratio of 1:1. After 1 h, the mixture was centrifuged at 1000× *g* for 15 min, the supernatant was discarded, and the resulting GS/Alg composite was stored at 4 °C.

**GS/Alg/Ca^2+^ constructs:** For rheological analysis, the GS/Alg composite was incubated in 3% (*w*/*v*) calcium chloride (CaCl_2_) solution at 4 °C for either 10 or 30 min, washed three times with PBS, and then placed at 37 °C overnight to ensure GS degradation. Parallel samples intended for scanning electron microscopy (SEM) imaging and cell loading into lyophilized gels were prepared using the same procedure, except that the incubation in CaCl_2_ solution was extended overnight.

**G/Alg/Ca^2+^ constructs:** The G/Alg mixture (4% (*w*/*v*) G, 1% (*w*/*v*) Alg) was immersed in a 3% (*w*/*v*) CaCl_2_ solution at 4 °C overnight. After washing three times with PBS, the samples were incubated overnight at 37 °C to ensure complete degradation of the G.

**GS/TN-Alg/Ca^2+^ constructs:** Following the same protocol as for GS/Alg/Ca^2+^ fabrication, a homogeneous blend of 2% (*w*/*v*) Tz- and Nb-Alg was mixed with an equal volume of GSs. After thorough mixing, the mixture was centrifuged to discard the supernatant, immersed in 3% (*w*/*v*) CaCl_2_ solution at 4 °C for 10 min, washed, and subsequently incubated overnight at 37 °C to ensure complete degradation of the GSs.

**GS/MA-Alg/BL constructs:** A 2% (*w*/*v*) MA-Alg solution containing 1% (*w*/*v*) LAP was mixed 1:1 (*v*/*v*) with GSs, incubated for 1 h, centrifuged, and the supernatant discarded. The GS/MA-Alg composite was blue light (BL)-crosslinked (405 nm, 4 °C, 10 min), washed with water, and incubated overnight at 37 °C.

It should be noted that for SEM characterization, GS/Alg/Ca^2+^ constructs were fabricated using both small (approx. 20–40 µm) and large (approx. 140–160 µm) GSs for comparison. GS/TN-Alg/Ca^2+^ constructs were also prepared with small (approx. 20–40 µm) GSs, similarly to the GS/Alg/Ca^2+^ constructs fabricated using microspheres of identical dimensions, for direct comparison. In all other instances, medium-diameter (approx. 60–90 µm) microspheres were used.

**Syringe-extruded GS-based constructs:** Using a syringe fitted with a 27G needle, GSs with average diameters of 8 μm, 20 μm, and 80 μm were extruded into filaments, and their morphologies were observed under an optical microscope. Additionally, a 1:1 (*v*/*v*) mixture of 20 μm GSs and 6% (*w*/*v*) MA-Alg (containing 1% (*w*/*v*) LAP) was extruded into filaments in the same manner, and its morphology was observed. Moreover, the GS/MA-Alg composite was extruded into culture medium using a syringe equipped with a 24G needle, followed by blue light crosslinking for 3 min. After 2 days of culture at 37 °C, the samples were freeze-dried and examined by SEM.

**Gelatin particle preparation via solvent-based method:** (1) Olive oil as oil phase: to 8 mL of olive oil containing 1% (*v*/*v*) Tween 80, 2 mL of 2% (*w*/*v*) Alg was added under stirring, and the mixture was stirred at 4 °C for 1 h. (2) Paraffin oil as oil phase: to 8 mL of paraffin oil containing 1% (*v*/*v*) Span 80, 2 mL of 2% (*w*/*v*) Alg was added under stirring, followed by stirring at 4 °C for 1 h. After stirring, the precipitate was collected by centrifugation, washed four times with 10 mL of isopropanol, and then washed three times with 10 mL of PBS.

### 4.5. Hydrogel Characterization

**Rheological characterization:** The viscoelasticity was measured using a rheometer (Anton Paar MCR 302, Graz, Austria) with an 25 mm diameter plate geometry. Amplitude sweeps were performed at 1.0 Hz. Frequency sweeps were conducted at a constant shear strain of 0.5%. The storage modulus (*G′*) and loss modulus (*G″*) were recorded and analyzed. The viscosity profiles were measured at shear rates from 0.1 to 1000 s^−1^, and stress relaxation tests were performed at a shear strain of 1%. Shear stress as a function of shear rate was measured across a sweep range of 0.01 to 100 s^−1^.

**Comparison of the effects of two freeze-drying methods on pore morphology:** 160 µm GS/Alg/Ca^2+^ hydrogels were subjected to either slow freezing at −80 °C for 4 h or flash freezing in liquid nitrogen for 8 min, followed by simultaneous lyophilization for SEM. Note: Except for this experiment, all other lyophilized samples were prepared by slow freezing at −80 °C.

**Morphological observation:** The lyophilized samples were sputter-coated with gold, and observed by SEM (Hitachi TM4000PLUS II, Tokyo, Japan).

**Confocal microscopy analysis:** 80 µm GS/GF-Alg/Ca^2+^ hydrogels (8 mm diameter × 2.5 mm height) were prepared similarly to unlabeled GS/Alg/Ca^2+^ hydrogels. Porosity was calculated from their porous morphology, and the porous structure was reconstructed into a 3D architecture (Leica STELLARIS 5, Wetzlar, Germany).

**Permeability test:** 80 µm GS/GF-Alg/Ca^2+^ hydrogels (8 mm diameter × 2.5 mm height) were incubated in 0.6% (*w*/*v*) Cy3-gelatin containing medium for 12 h to assess the fraction of pores penetrated by Cy3-gelatin.

**Time-dependent pore formation analysis:** Similar to the unlabeled samples, 80 µm Cy3-GS was mixed with Alg, crosslinked with CaCl_2_ solution overnight at 4 °C. After the CaCl_2_ solution was removed, the constructs (8 mm diameter × 5 mm height) were immersed in 1.5 mL of 0.9% (*w*/*v*) NaCl solution at 37 °C. At 0.5, 2, 4, 8, 12, and 24 h, 0.15 mL of the solution was withdrawn from each sample and replaced with fresh solution. The Cy3 fluorescence intensities were measured using a microplate reader (Thermo Scientific Varioskan LUX, Waltham, MA, USA). Additionally, the internal morphology of the constructs was recorded under an optical microscope after 0 and 4 h of culture at 37 °C.

### 4.6. Preparation of Cell-Laden Hydrogels

Figure S10 depicts the main workflow for cell-laden sample preparation:

**G/Alg/Ca^2+^/cells, GS/Alg/Ca^2+^/cells, and GS/MA-Alg/BL/cells constructs:** The lyophilized scaffolds (G/Alg/Ca^2+^, 80 µm GS/Alg/Ca^2+^, and 80 µm GS/MA-Alg/BL constructs) were first prepared. Each scaffold was then seeded with 2 × 10^7^ EGFP-Jurkat cells in 80 μL of medium. After the cell suspensions were fully absorbed, the constructs were rehydrated in culture medium and incubated. Jurkat cells harvested from GS/Alg/Ca^2+^/cells constructs on day 3 were stimulated with CD3/CD28 magnetic beads for 24 h, followed by double staining with Fixable Viability Dye and anti-CD25 antibody. Cells from GS/MA-Alg/BL/cells constructs were collected on day 6 for the same stimulation and double staining, and additionally on day 17 for APC-Annexin V/7-AAD staining.

**GS/Alg/Ca^2+^ (cells) constructs:** A suspension of Jurkat cells (1 × 10^7^/mL) in 80 µm GSs was combined with an equal volume of 2% (*w*/*v*) Alg solution, crosslinked in 3% (*w*/*v*) CaCl_2_ solution for 5 min at 4 °C, washed with PBS, and subsequently cultured. On day 3, cells were harvested and subjected to 7-AAD staining and CTV labeling.

**G/Alg/Ca^2+^ (cells) constructs:** Jurkat cells (1 × 10^7^/mL) were resuspended in an 8% (*w*/*v*) G solution and then mixed with an equal volume of 2% (*w*/*v*) Alg solution. Subsequent procedures were identical to those described for the GS/Alg/Ca^2+^ (cells) group.

**Syringe-extruded cell-laden GS/MA-Alg/BL constructs:** A 1:1 (*v*/*v*) blend of 20–30 µm GSs (loaded with 6 × 10^7^/mL EGFP-Jurkat cells) and 6% (*w*/*v*) MA-Alg (containing 1% (*w*/*v*) LAP) was extruded through a 24G syringe, crosslinked under 405 nm blue light for 1 min, and then cultured for 21 h. Subsequently, cell morphology and EGFP fluorescence were evaluated.

### 4.7. Characterization of Cell-Laden Hydrogels

**APC-Annexin V/7-AAD double staining:** For each sample, cells (3 × 10^5^) were pelleted (300× *g*, 5 min), washed twice, and resuspended in 40 µL of Annexin V Binding Buffer. After staining with 2 µL each of APC-Annexin V and 7-AAD (20 min, room temperature, dark), 400 µL of buffer was added, and the cells were filtered for flow cytometry (BD LSRFortessa^TM^, Franklin Lakes, NJ, USA).

**7-AAD staining:** For each sample, cells (3 × 10^5^) were pelleted (300× *g*, 5 min), washed once with PBS, and resuspended in 500 µL of PBS. After staining with 5 µL of 7-AAD (20 min, room temperature, dark), the cells were filtered prior to flow cytometric analysis.

**Fixable Viability Dye/anti-human CD25 antibody double staining:** For each sample, cells (3 × 10^5^) were pelleted (300× *g*, 5 min), leaving about 20 µL residual volume. Samples were stained with 1 µL of anti-human CD25 antibody (20 min, room temperature, dark), washed with PBS, then incubated with 50 µL of 1:1000 diluted Fixable Viability Dye (15 min, room temperature, dark). After a final PBS wash, cells were filtered and analyzed by flow cytometry.

**CTV labeling:** For each sample, cells (6 × 10^5^) were washed with PBS, resuspended in 1 mL PBS, and stained with 1 µL of 1:10 diluted CTV (37 °C, 7 min, dark). Staining was quenched with complete medium (room temperature, 5 min, dark). After centrifugation and resuspension, half of the cells were cultured for 48 h and half were immediately analyzed by flow cytometry for baseline CTV fluorescence. The cultured cells were then analyzed for CTV dilution, and the proliferation indices were calculated using FlowJo software 10.8.1.

**Confocal microscopy analysis:** Cy3-GS/GF-Alg/Ca^2+^ (DAPI-cells) hydrogels were fabricated using the same method as GS/Alg/Ca^2+^ (cells) hydrogels and stored at 4 °C to visualize the distribution of the three fluorescent markers by confocal microscope system (Andor Dragonfly200, Belfast, UK). After washing and incubation at 37 °C for 48 h, the distribution was re-evaluated. 

### 4.8. Statistical Analysis

Statistical data analysis was performed using the GraphPad prism 6, and *p* values < 0.05 were considered statistically significant. * *p* < 0.05, ** *p* < 0.01, *** *p* < 0.001, **** *p* < 0.0001. Confocal images were processed using ImarisViewer 10.2.0. Image J was used to measure GSs size and hydrogel porosity.

## Data Availability

The original contributions presented in this study are included in the article/Supplementary Material. Further inquiries can be directed to the corresponding author.

## Supplementary Materials

The following supporting information can be downloaded at: https://www.mdpi.com/article/10.3390/gels12060477/s1. Table S1: GSs average diameters and polydispersity indices from three replicate batches prepared with different formulations; Figure S1: Morphology of gelatin particles produced via the solvent-based method; Figure S2: Rheological properties of gelatin microspheres; Figure S3: Comparison of ^1^H NMR spectra between click-modified and unmodified alginate; Figure S4: Structural morphology of hydrogels constructed from gelatin microspheres; Figure S5: Morphological comparison of porous GS/Alg/Ca^2+^ hydrogels fabricated via two freeze-drying approaches; Figure S6: Morphological characterization of GS-based porous hydrogels using confocal microscopy; Figure S7: Time-dependent pore formation within Cy3-GS/Alg/Ca^2+^ constructs; Figure S8: Optical microscopy images of EGFP-Jurkat cell-loaded GS/MA-Alg/BL hydrogel microfibers; Figure S9: Fabrication strategy for porous hydrogels based on gelatin microspheres/(modified) alginate composites; Figure S10: Two strategies for cell loading using gelatin microspheres/(modified) alginate composites-based porous hydrogels.

## References

[1] Teng Y., Chi J., Huang J., Li Z., Li S., Wu X., Zhu L., Ren J. (2025). Hydrogel toughening resets biomedical application boundaries. Prog. Polym. Sci..

[2] Sui J., Pragnere S., Kurniawan N.A. (2025). Revisiting the biophysical aspects of extracellular-matrix-mimicking hydrogels: What cells see vs. what cells feel. Biomater. Sci..

[3] Bordbar-Khiabani A., Gasik M. (2022). Smart hydrogels for advanced drug delivery systems. Int. J. Mol. Sci..

[4] Puiggalí-Jou A., Hui I.B., Fernandez-Rico C., Zenobi-Wong M. (2026). The space within: How architected voids promote tissue formation. Adv. Mater..

[5] Mirmusavi M.H., Gonçalves V.S.S., Wischke C. (2026). Gelatin-based porous scaffolds: Design concepts, production, and applications in precision regenerative medicine. Mater. Today Bio.

[6] Wu C., Zhang H., Guo Y., Sun X., Hu Z., Teng L., Zeng Z. (2024). Porous hydrogels for immunomodulatory applications. Int. J. Mol. Sci..

[7] Liu Y., Ling S., Chen Z., Xu J. (2025). Recent advances of macroporous hydrogel microparticles: Fabrication and applications. Chem. Eng. J..

[8] Li H., Yu L., Li Z., Li S., Liu Y., Qu G., Chen K., Huang L., Li Z., Ren J. (2025). A narrative review of bioactive hydrogel microspheres: Ingredients, modifications, fabrications, biological functions, and applications. Small.

[9] Wang Z., Cai Y., Yuan K., Zhang L., Xiong R., Zhuang Z., Huang C. (2025). Bio-based multicompartmental microspheres: From structural innovation to precision biomedicine. Chem. Eng. J..

[10] Wang Z., Lin Z., Mei X., Cai L., Lin K., Rodríguez J., Ye Z., Parraguez X.S., Guajardo E.M., Luna P.C.G. (2025). Engineered living systems based on gelatin: Design, manufacturing, and applications. Adv. Mater..

[11] Dai W., Luo Y., Zhou K., Wu Z., Jian X., Xu H., He W., Zhang D. (2026). Microfluidics-based hydrogel microspheres: Complex structure design and multidisciplinary applications. Chem. Eng. Process..

[12] Valente K., Boice G.N., Polglase C., Belli R.G., Bourque E., Suleman A., Brolo A. (2024). Synthesis of gelatin methacryloyl analogs and their use in the fabrication of pH-responsive microspheres. Pharmaceutics.

[13] Yang X., Xu Y., Zhu D., Mi X. (2025). GelMA core-shell microgel preparation based on a droplet microfluidic device for three-dimensional tumor ball culture and its drug testing. Molecules.

[14] Lau T.T., Lee L.Q.P., Leong W., Wang D.-A. (2012). Formation of model hepatocellular aggregates in a hydrogel scaffold using degradable genipin crosslinked gelatin microspheres as cell carriers. Biomed. Mater..

[15] Wang L., Lu S., Lam J., Kasper F.K., Mikos A.G. (2015). Fabrication of cell-laden macroporous biodegradable hydrogels with tunable porosities and pore sizes. Tissue Eng. Part C Methods.

[16] Wang E., Wang D., Geng A., Seo R., Gong X. (2017). Growth of hollow cell spheroids in microbead templated chambers. Biomaterials.

[17] Demir B., Hacker M.C. (2025). Critical process parameters of gelatin microparticle fabrication with a water-in-oil emulsion method. Int. J. Pharm..

[18] Lee A., Hudson A.R., Shiwarski D.J., Tashman J.W., Hinton T.J., Yerneni S., Bliley J.M., Campbell P.G., Feinberg A.W. (2019). 3D bioprinting of collagen to rebuild components of the human heart. Science.

[19] Wu C.A., Zhu Y., Venkatesh A., Stark C.J., Lee S.H., Woo Y.J. (2023). Optimization of freeform reversible embedding of suspended hydrogel microspheres for substantially improved three-dimensional bioprinting capabilities. Tissue Eng. Part C Methods.

[20] Seymour A.J., Shin S., Heilshorn S.C. (2021). 3D printing of microgel scaffolds with tunable void fraction to promote cell infiltration. Adv. Healthc. Mater..

[21] Hudson A.R., Shiwarski D.J., Kramer A.J., Feinberg A.W. (2025). Enhancing viability in static and perfused 3D tissue constructs using sacrificial gelatin microparticles. ACS Biomater. Sci. Eng..

[22] Suhoski M.M., Golovina T.N., Aqui N.A., Tai V.C., Varela-Rohena A., Milone M.C., Carroll R.G., Riley J.L., June C.H. (2007). Engineering artificial antigen-presenting cells to express a diverse array of co-stimulatory molecules. Mol. Ther..

[23] Shou X., Zhang H., Wu D., Zhong L., Ni D., Kong T., Zhao Y., Zhao Y. (2021). Antigen-presenting hybrid colloidal crystal clusters for promoting T cells expansion. Small.

[24] Thaventhiran J.E.D., Hoffmann A., Magiera L., de la Roche M., Lingel H., Brunner-Weinzierl M., Fearon D.T. (2012). Activation of the hippo pathway by CTLA-4 regulates the expression of blimp-1 in the CD8+ T cell. Proc. Natl. Acad. Sci. USA.

[25] Nickerson M.T., Patel J., Heyd D.V., Rousseau D., Paulson A.T. (2006). Kinetic and mechanistic considerations in the gelation of genipin-crosslinked gelatin. Int. J. Biol. Macromol..

[26] Attwood D., Collett J.H., Tait C.J. (1985). The micellar properties of the poly(oxyethylene)-poly(oxypropylene) copolymer pluronic F127 in water and electrolyte solution. Int. J. Pharm..

[27] Malektaj H., Drozdov A.D., deClaville Christiansen J. (2023). Swelling of homogeneous alginate gels with multi-stimuli sensitivity. Int. J. Mol. Sci..

[28] Agarwalla P., Ogunnaike E.A., Ahn S., Froehlich K.A., Jansson A., Ligler F.S., Dotti G., Brudno Y. (2022). Bioinstructive implantable scaffolds for rapid in vivo manufacture and release of CAR-T cells. Nat. Biotechnol..

[29] Bao P., Gu H.-Y., Jiang Y.-C., Wang J.-W., Wu M., Yu A., Zhong Z., Zhang X.-Z. (2024). In situ sprayed exosome-cross-linked gel as artificial lymph nodes for postoperative glioblastoma immunotherapy. ACS Nano.

[30] Vining K.H., Marneth A.E., Adu-Berchie K., Grolman J.M., Tringides C.M., Liu Y., Wong W.J., Pozdnyakova O., Severgnini M., Stafford A. (2022). Mechanical checkpoint regulates monocyte differentiation in fibrotic niches. Nat. Mater..

[31] Liu Z., Li Y.-R., Yang Y., Zhu Y., Yuan W., Hoffman T., Wu Y., Zhu E., Zarubova J., Shen J. (2024). Viscoelastic synthetic antigen-presenting cells for augmenting the potency of cancer therapies. Nat. Biomed. Eng..

[32] Soares D.J., McCarthy A.D. (2024). The impact of gel parameters on the dispersal and fragmentation of hyaluronic acid gel fillers within an artificial model of arterial embolism. Gels.

[33] Nam S., Stowers R., Lou J., Xia Y., Chaudhuri O. (2019). Varying PEG density to control stress relaxation in alginate-PEG hydrogels for 3D cell culture studies. Biomaterials.

[34] Cacopardo L., Guazzelli N., Nossa R., Mattei G., Ahluwalia A. (2019). Engineering hydrogel viscoelasticity. J. Mech. Behav. Biomed. Mater..

[35] Huang J., Fu H., Wang Z., Meng Q., Liu S., Wang H., Zheng X., Dai J., Zhang Z. (2016). BMSCs-laden gelatin/sodium alginate/carboxymethyl chitosan hydrogel for 3D bioprinting. RSC Adv..

[36] Zmora S., Glicklis R., Cohen S. (2002). Tailoring the pore architecture in 3-D alginate scaffolds by controlling the freezing regime during fabrication. Biomaterials.

[37] Van Vlierberghe S., Cnudde V., Dubruel P., Masschaele B., Cosijns A., De Paepe I., Jacobs P.J.S., Van Hoorebeke L., Remon J.P., Schacht E. (2007). Porous gelatin hydrogels: 1. cryogenic formation and structure analysis. Biomacromolecules.

[38] Liu R., Jia L., Chen J., Long Y., Zeng J., Liu S., Pan B., Liu X., Jiang H. (2024). Chondrocyte spheroid-laden microporous hydrogel-based 3D bioprinting for cartilage regeneration. Int. J. Bioprinting.

[39] Xiang Z., Chen H., Xu B., Wang H., Zhang T., Guan X., Ma Z., Liang K., Shi Q. (2024). Gelatin/heparin coated bio-inspired polyurethane composite fibers to construct small-caliber artificial blood vessel grafts. Int. J. Biol. Macromol..

[40] Guo Y., Han Y., Cao Y., Chen Y., Xie J., Ding H., Liang S., Liu X., Sun W., Tang J. (2025). Facile fabrication of tough super macroporous hydrogel via enhanced phase separation. Adv. Funct. Mater..

[41] Torres-Sanchez C., Al Mushref F.R.A., Norrito M., Yendall K., Liu Y., Conway P.P. (2017). The effect of pore size and porosity on mechanical properties and biological response of porous titanium scaffolds. Mater. Sci. Eng. C.

[42] Hinton T.J., Jallerat Q., Palchesko R.N., Park J.H., Grodzicki M.S., Shue H.-J., Ramadan M.H., Hudson A.R., Feinberg A.W. (2015). Three-dimensional printing of complex biological structures by freeform reversible embedding of suspended hydrogels. Sci. Adv..

[43] Desai R.M., Koshy S.T., Hilderbrand S.A., Mooney D.J., Joshi N.S. (2015). Versatile click alginate hydrogels crosslinked via tetrazine-norbornene chemistry. Biomaterials.

