# Supplementary material for: Facile and Green Fabrication of Porous Hydrogels Based on Gelatin Microsphere Porogens for 3D Immune Cell Culture
Source: Gels. 2026 May 29;12(6):477. doi: 10.3390/gels12060477 (PMC13297822; doi:10.3390/gels12060477)
Supplement: Supplementary file 1 [file gels-12-00477-s001.zip › gels-4281146-supplementary.pdf]

Supporting Information

Facile and Green Fabrication of Porous Hydrogels Based on Gelatin Microsphere Porogens for 3D Immune Cell Culture

Han Fu [1,2], Qiwen Yao [1,2], Shuai Tan [1,2], Yingming Wang [1,2] and Aishun Jin [1,2,*]

[1] Department of Immunology, School of Basic Medical Sciences, Chongqing Medical University, Chongqing 400010, China; f815819120@126.com (H.F.); 2023110070@stu.cqmu.edu.cn (Q.Y.); 2023110068@stu.cqmu.edu.cn (S.T.); wangyingming0908@cqmu.edu.cn (Y.W.)

[2] Chongqing Key Laboratory of Tumor Immune Regulation and Immune Intervention, Chongqing 400010, China

* Correspondence: aishunjin@cqmu.edu.cn

Table S1. GSs average diameters and polydispersity indices from three replicate batches prepared with different formulations.

Group	Batch 1		Batch 2		Batch3	
	Diameter (μm)	PDI[a]	Diameter (μm)	PDI	Diameter (μm)	PDI
0.5% (w/v) Pluronic®F-127	48.99 ± 8.29	0.03	33.77 ± 6.20	0.03	33.73 ± 5.98	0.03
1.25% (w/v) Pluronic®F-127	62.39 ± 11.24	0.03	61.32 ± 8.63	0.02	72.03 ± 10.91	0.02
2.5% (w/v) Pluronic®F-127	116.78 ± 21.93	0.04	85.66 ± 13.48	0.02	82.10 ± 14.92	0.03
Vwater:Vethanol=1:1.18	38.84 ± 6.75	0.03	29.11 ± 4.97	0.03	40.96 ± 4.81	0.01
Vwater:Vethanol=1:1.36	74.22 ± 10.83	0.02	96.92 ± 15.21	0.02	60.74 ± 6.72	0.01
Vwater:Vethanol=1:1.55	153.25 ± 29.93	0.04	149.42 ± 30.64	0.04	165.94 ± 31.43	0.04
pH c=pH 10.0	23.93 ± 5.32	0.05	24.25 ± 4.82	0.04	32.02 ± 8.79	0.08
T d°C=24°C	223.75 ± 41.30	0.03	234.76 ± 34.57	0.02	260.72 ± 53.63	0.04
Ultra-small GS	7.54 ± 1.69	0.05	7.37 ± 1.87	0.06	9.90 ± 2.34	0.06

a: Polydispersity index, PDI

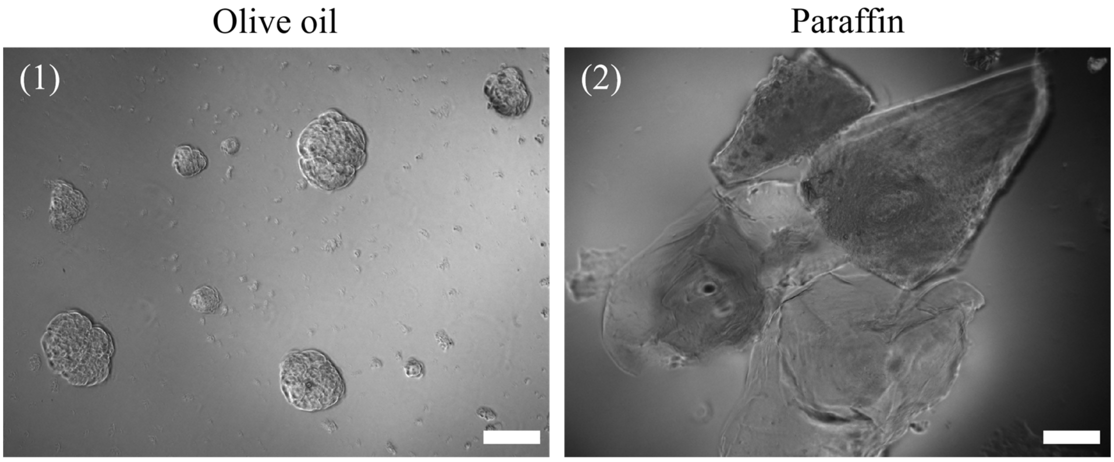

Figure S1. Morphology of gelatin particles produced via the solvent-based method. (1) Olive oil and (2) paraffin as the oil-phase solvent. Scale bar: 250 μm.

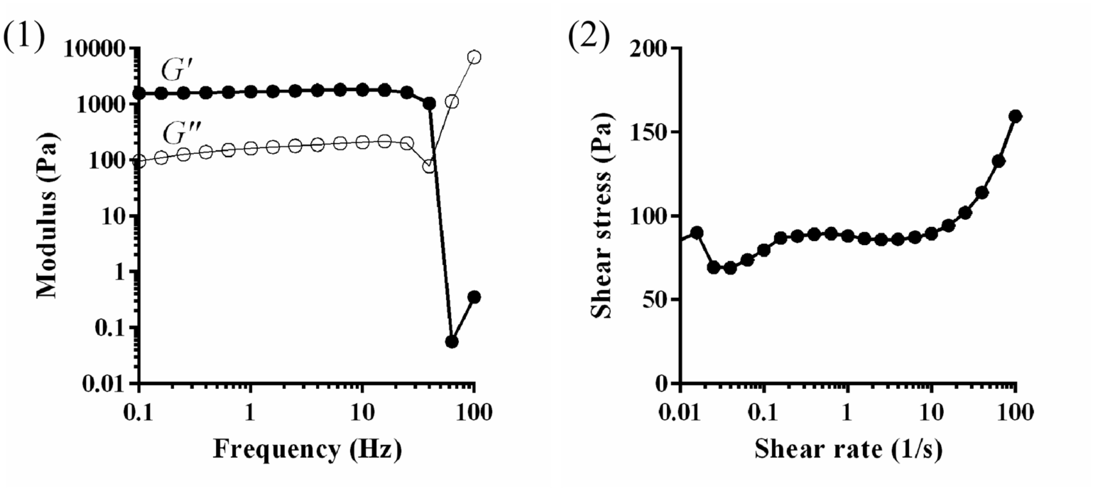

Figure S2. Rheological properties of gelatin microspheres. (1) Frequency sweep curves and (2) shear stress curve of gelatin microspheres.

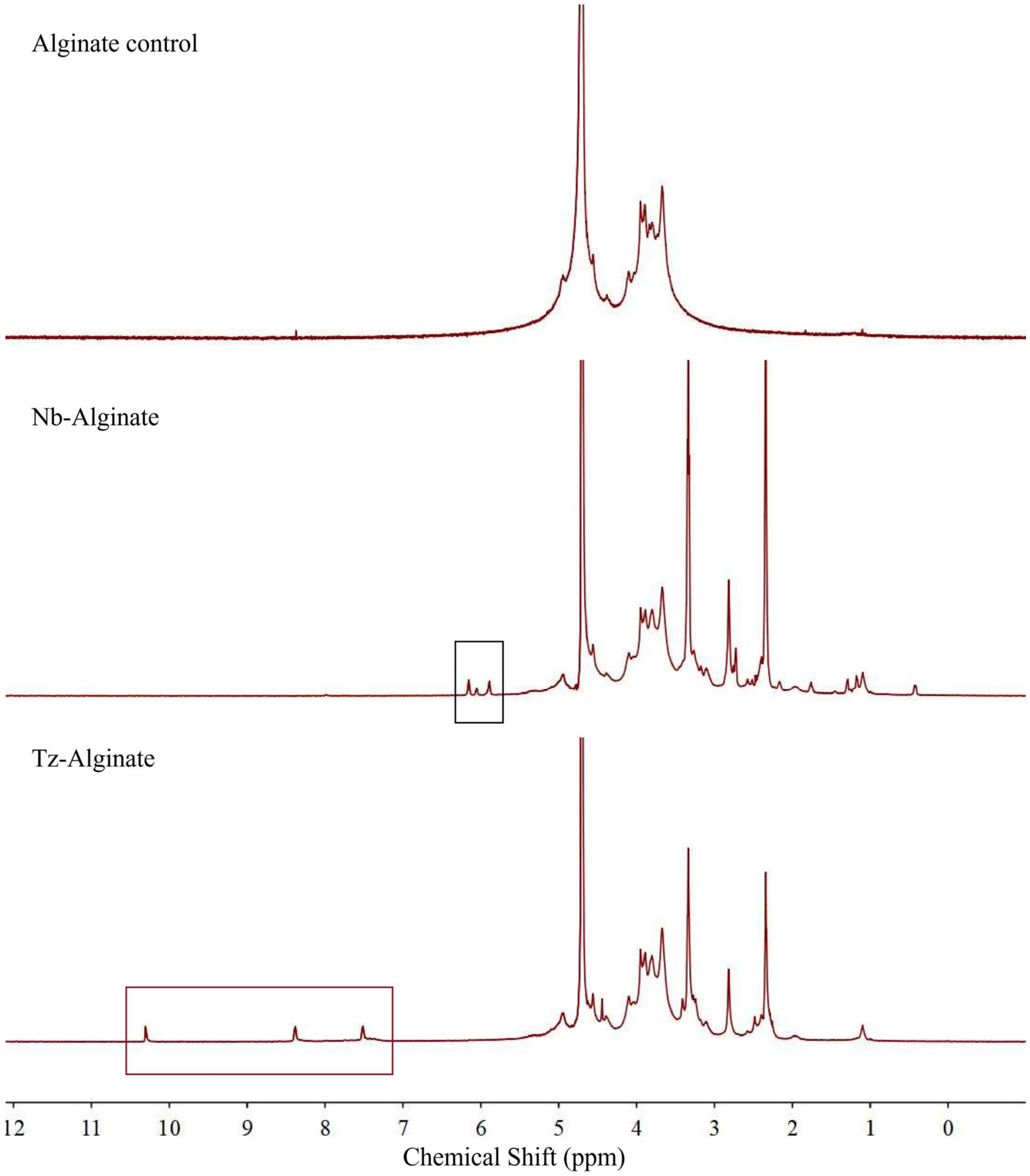

Figure S3. Comparison of ^{1}H NMR spectra between click-modified and unmodified alginate. The functionalized alginate was prepared by covalent coupling of either Nb (5-(aminomethyl)bicyclo[2.2.1]hept-2-ene, Nb-Alginate) or Tz ((4-(1,2,4,5-tetrazin-3-yl)phenyl)methanamine hydrochloride, Tz-Alginate). The black and red boxes indicate the alkene protons (Nb-Alginate) and aromatic protons (Tz-Alginate), respectively, confirming successful norbornene and tetrazine conjugation.

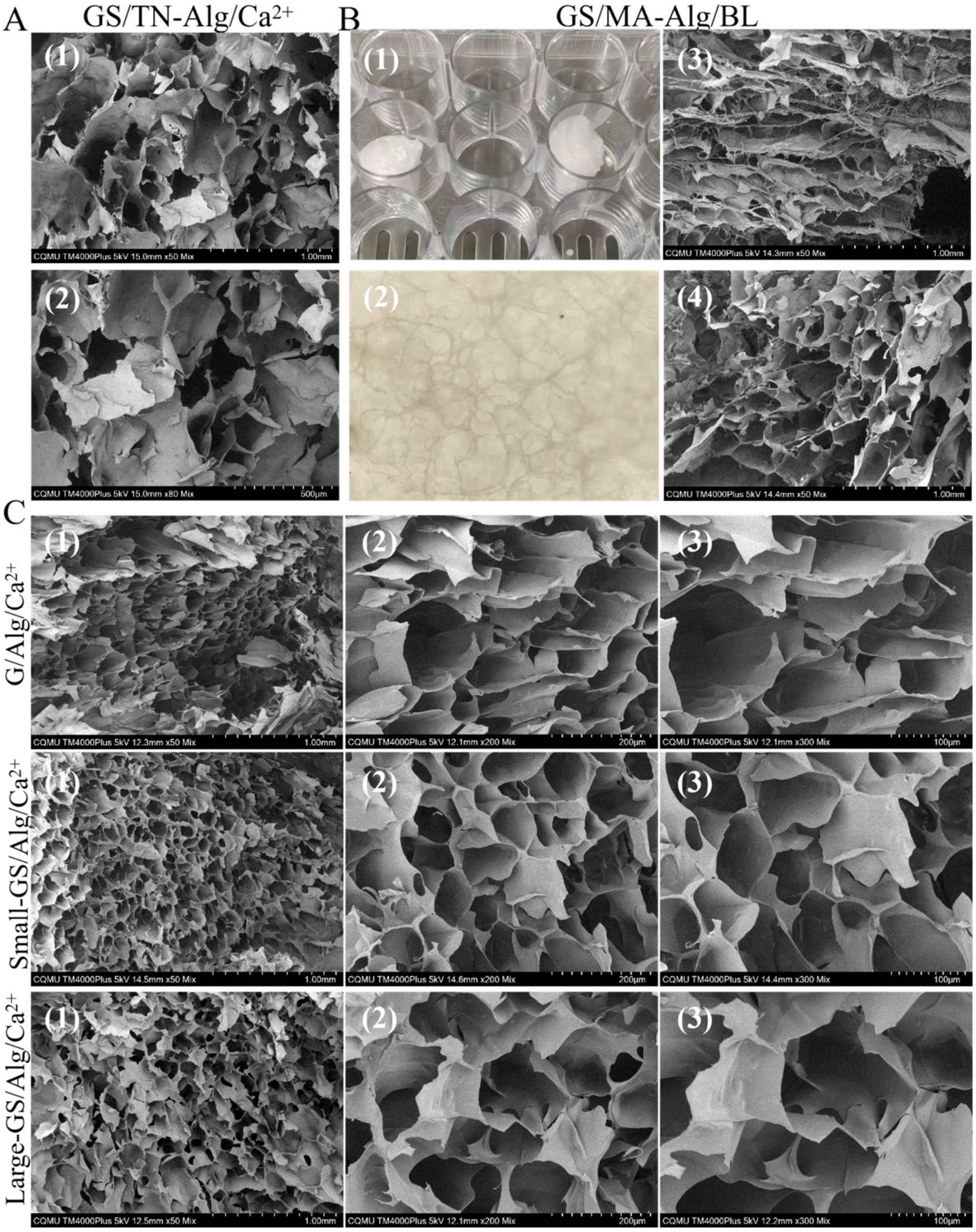

Figure S4. Structural morphology of hydrogels constructed from gelatin microspheres. Abbreviations: G, gelatin; GS, gelatin microspheres; Alg, alginate; Ca^{2+}, calcium ion; TN-Alg, a mixture of tetrazine (Tz)- and norbornene (Nb)-modified Alg; MA-Alg, methacrylated alginate; BL, blue light; SEM, scanning electron microscopy. A (1, 2) SEM images at two different magnifications of GS/TN-Alg/Ca^{2+} hydrogel. B (1) Lyophilized samples of GS/MA-Alg/BL hydrogels. (2) Optical microscopy image of lyophilized GS/MA-Alg/BL hydrogel. (3, 4) SEM images of two different cross-sections of the GS/MA-Alg/BL hydrogel. C SEM images at different magnifications of three Ca^{2+}-crosslinked hydrogels: (1) G/Alg/Ca^{2+}, (2) small-diameter GS/Alg/Ca^{2+}, and (3) large-diameter GS/Alg/Ca^{2+}.

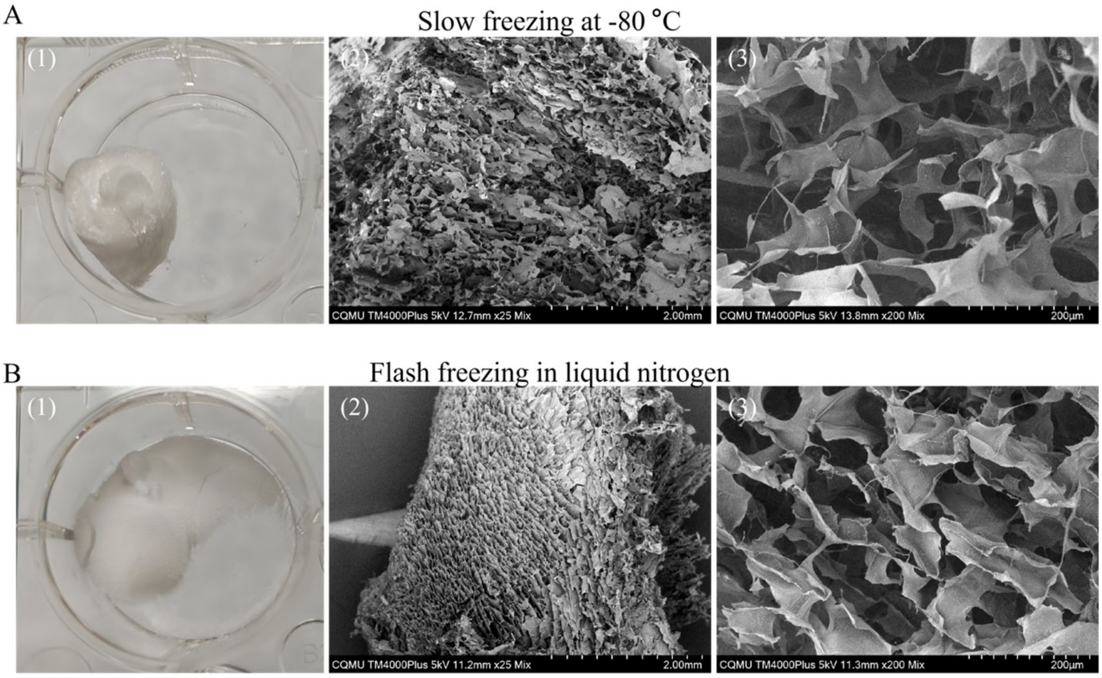

Figure S5. Morphological comparison of porous GS/Alg/Ca^{2+} hydrogels fabricated via two freeze-drying approaches. Abbreviations: GS, gelatin microspheres; Alg, alginate; Ca^{2+}, calcium ion; SEM, scanning electron microscopy. (A) Slow freezing at -80 °C: (1) appearance, (2) low-magnification SEM, and (3) high-magnification SEM. (B) Flash freezing in liquid nitrogen: (1) appearance, (2) low-magnification SEM, and (3) high-magnification SEM.

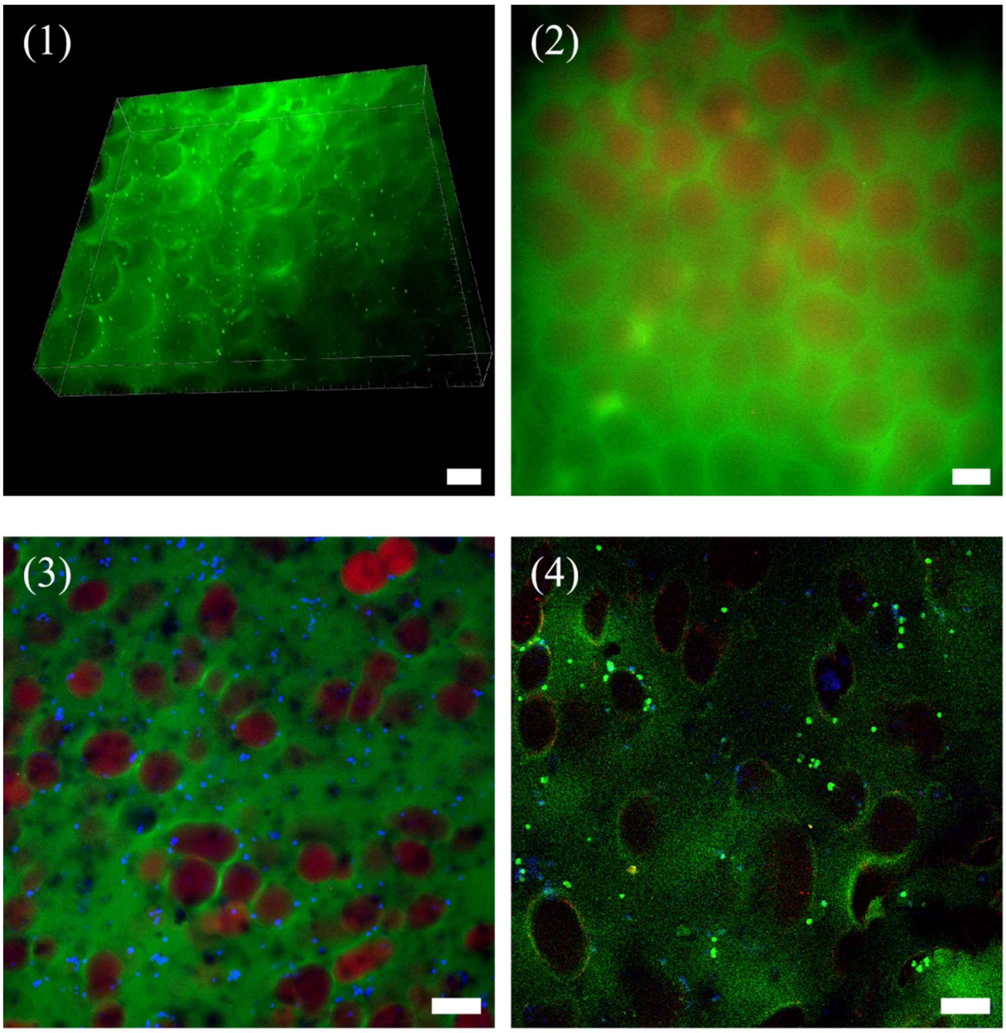

Figure S6. Morphological characterization of GS-based porous hydrogels using confocal microscopy. Abbreviations: GS, gelatin microspheres; GF-Alg, green fluorescence-labeled Alg; Ca^{2+}, calcium ion. (1) 3D reconstructed image of a GS/GF-Alg/Ca^{2+} hydrogel. Green: GF-Alg. (2) Permeability test image of a GS/GF-Alg/Ca^{2+} hydrogel. Green: GF-Alg. Red: Cy3-gelatin. (3) Morphology of a Cy3-GS/GF-Alg/Ca^{2+}(DAPI-cells) hydrogel stored at 4 °C. Green: GF-Alg; Red: Cy3-GS; Blue: DAPI-cells. (4) Morphology of a Cy3-GS/GF-Alg/Ca^{2+}(DAPI-cells) hydrogel after incubation at 37 °C. Green: GF-Alg; Red: Cy3-gelatin; Blue: DAPI-cells. Scale bar: 100 μm.

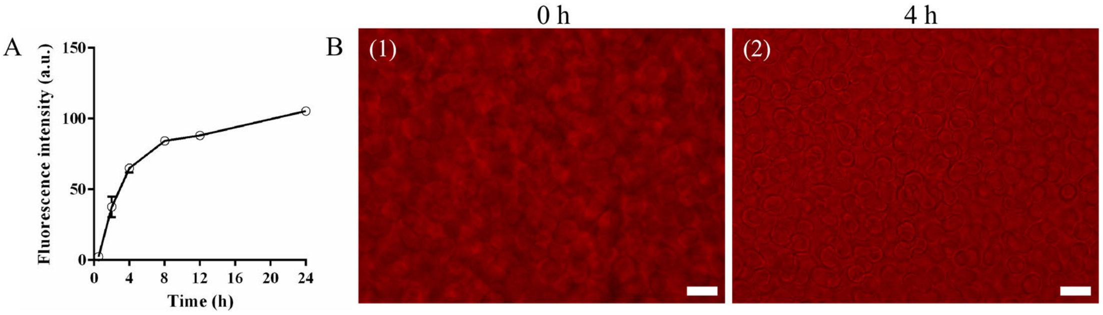

Figure S7. Time-dependent pore formation within Cy3-GS/Alg/Ca^{2+} constructs. Abbreviations: GS, gelatin microspheres; Alg, alginate; Ca^{2+}, calcium ion. (A) Release kinetics of Cy3-labeled GS from constructs incubated at 37 °C. (B) Optical micrographs of the internal morphology of the constructs after (1) 0 and (2) 4 h culture at 37°C. Red: (1) Cy3-GS, (2) Cy3-gelatin. Scale bar: 100 μm.

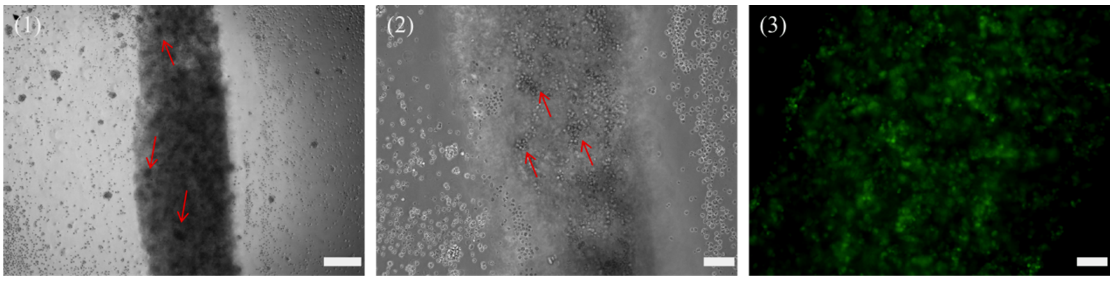

Figure S8. Optical microscopy images of EGFP-Jurkat cell-loaded GS/MA-Alg/BL hydrogel microfibers. (1) 5× and (2) 10× bright-field, and (3) 10× fluorescence. Abbreviations: GS, gelatin microspheres; MA-Alg, methacrylated alginate; BL, blue light. Scale bars: (1) 250 μm, (2) 100 μm, (3) 100 μm. Red arrows indicate cell aggregates.

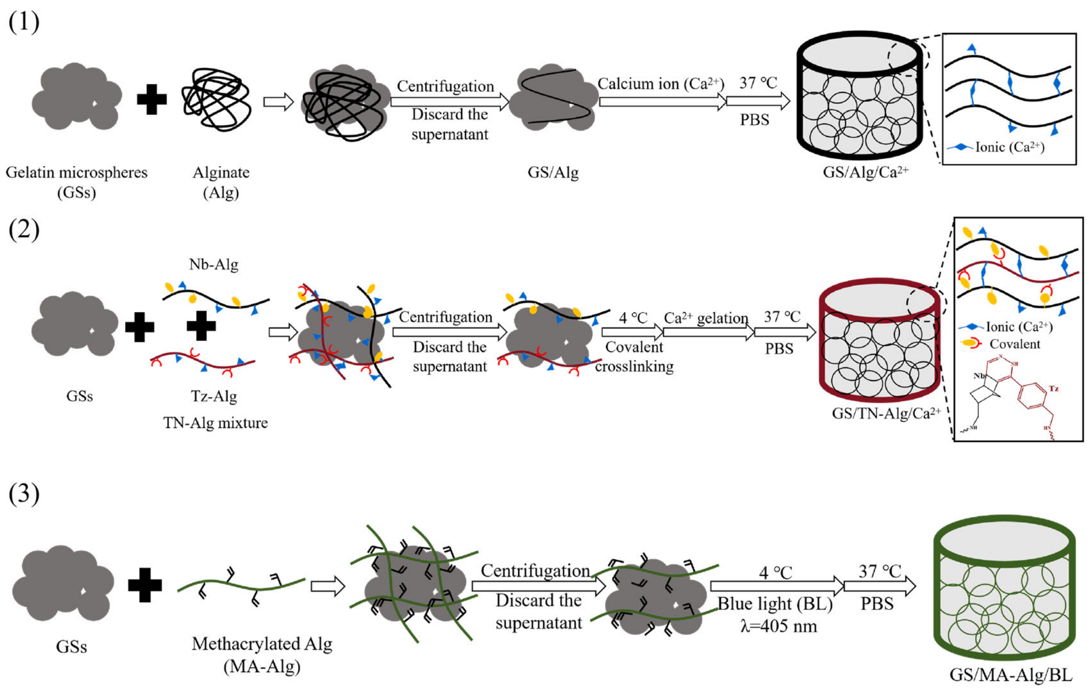

Figure S9. Fabrication strategy for porous hydrogels based on gelatin microspheres/(modified) alginate composites. Abbreviations: GS, gelatin microspheres; Alg, alginate; Ca^{2+}, calcium ion; TN-Alg, a mixture of tetrazine (Tz)- and norbornene (Nb)-modified Alg; MA-Alg, methacrylated alginate; BL, blue light. Fabrication process: (1) Ca^{2+}-crosslinked viscous GS/Alg/Ca^{2+} hydrogels; (2) dual-crosslinked elastic GS/TN-Alg/Ca^{2+} hydrogels; (3) photo-crosslinked GS/MA-Alg/BL hydrogels.

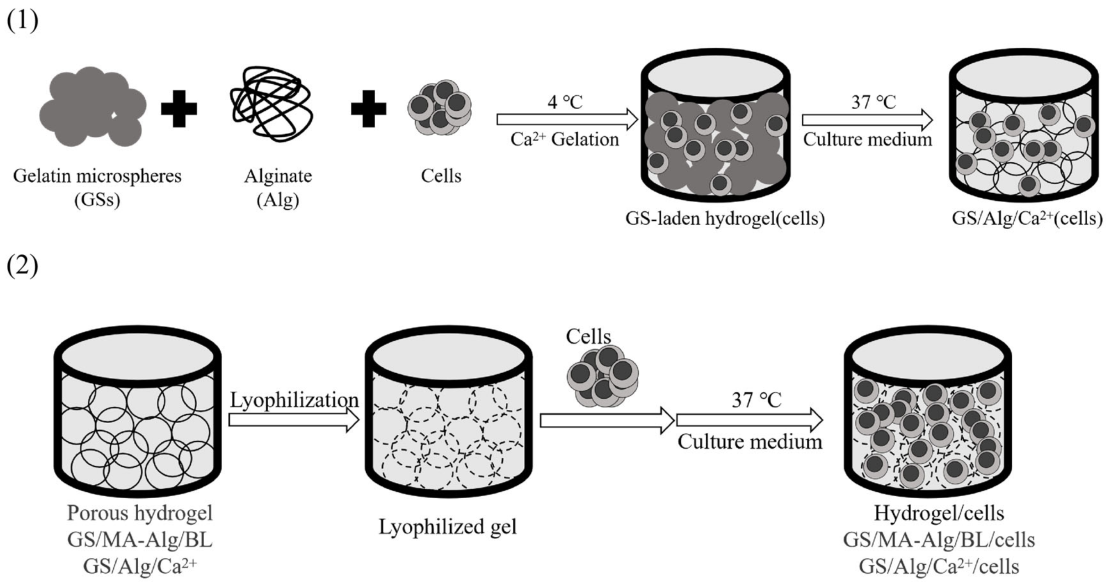

Figure S10. Two strategies for cell loading using gelatin microspheres/(modified) alginate composites-based porous hydrogels. Abbreviations: GS, gelatin microspheres; Alg, alginate; MA-Alg, methacrylated alginate; Ca^{2+}, calcium ion; BL, blue light. (1) Jurkat cells were encapsulated in situ using GS/Alg composites, crosslinked with Ca^{2+}, and then incubated at 37 °C to degrade the GS, allowing the cells to be cultured within the resulting porous hydrogel (GS/Alg/Ca^{2+}(cells)). (2) Photo-crosslinked hydrogels (GS/MA-Alg/BL) and Ca^{2+}-crosslinked hydrogels (GS/Alg/Ca^{2+}) were freeze-dried, followed by adsorption of Jurkat cells into the pores and subsequent re-swelling in culture medium (Hydrogel/cells: GS/MA-Alg/BL/cells, GS/Alg/Ca^{2+}/cells).
